# Genotype Pattern Mining for Pairs of Interacting Variants Underlying Digenic Traits

**DOI:** 10.3390/genes12081160

**Published:** 2021-07-28

**Authors:** Atsuko Okazaki, Sukanya Horpaopan, Qingrun Zhang, Matthew Randesi, Jurg Ott

**Affiliations:** 1 Department of Diagnostics and Therapeutics of Intractable Diseases, Juntendo University, Bunkyo-ku, Tokyo 113-8421, Japan; a-okazaki@juntendo.ac.jp; 2 Laboratory of Statistical Genetics, Rockefeller University, New York, NY 10065, USA; 3 Department of Anatomy, Faculty of Medical Science, Naresuan University, Phitsanulok 65000, Thailand; sukanyahor@gmail.com; 4 Department of Mathematics and Statistics, University of Calgary, Calgary, AB T2N 1N4, Canada; qingrun.zhang@ucalgary.ca; 5 Laboratory of the Biology of Addictive Diseases, Rockefeller University, New York, NY 10065, USA; matthew.randesi@rockefeller.edu

**Keywords:** pattern mining, digenic traits, genotype pattern, diplotype

## Abstract

Some genetic diseases (“digenic traits”) are due to the interaction between two DNA variants, which presumably reflects biochemical interactions. For example, certain forms of Retinitis Pigmentosa, a type of blindness, occur in the presence of two mutant variants, one each in the *ROM1* and *RDS* genes, while the occurrence of only one such variant results in a normal phenotype. Detecting variant pairs underlying digenic traits by standard genetic methods is difficult and is downright impossible when individual variants alone have minimal effects. Frequent pattern mining (FPM) methods are known to detect patterns of items. We make use of FPM approaches to find pairs of genotypes (from different variants) that can discriminate between cases and controls. Our method is based on genotype patterns of length two, and permutation testing allows assigning *p*-values to genotype patterns, where the null hypothesis refers to equal pattern frequencies in cases and controls. We compare different interaction search approaches and their properties on the basis of published datasets. Our implementation of FPM to case-control studies is freely available.

## 1. Introduction

Over the years, various examples of the combined actions of two disease-causing variants (single-nucleotide polymorphisms, SNPs) have been published (digenic inheritance [1,2]). For example, certain forms of Retinitis Pigmentosa (genetic blindness) occur in the presence of two mutant variants, one each in the *ROM1* and *RDS* genes, while the occurrence of only one such variant results in a normal phenotype [3]. Some of these instances of digenic inheritance were found fortuitously. For example, in an investigation of a large family, five members with severe insulin resistance, and no other family members, were heterozygous at each of the two genes located on different chromosomes [4].

In statistical analysis of case-control studies, interaction has been defined as the extent and manner in which two causes of a disease modify the strength of one another, with different types like removable and essential interactions being distinguished [5]. A scholarly article on epistasis (gene-gene interaction), published in 2002, provided a historical background to epistatic interaction effects and a survey of some methods of epistasis detection [6]. More discussion from a statistical viewpoint may be found in newer papers [7,8].

It has long been recognized that interactions among loci contribute to disease, and strategies for searching for interactions have been discussed [9,10]. In the Methods section, we outline some approaches to the detection and analysis of epistatic interactions in genetics, with particular emphasis on data mining and machine learning methods [11]. A recent investigation of quantitative trait loci in mice significantly demonstrated multiple epistatic interactions, while single-locus analyses were far from significant [12], and large-scale investigations into epistatic effects on disease phenotypes are currently underway [13].

Different methods are often compared on the basis of computer-generated data that are obtained with suitable interaction models between DNA variants and disease phenotypes. While such data allow for power calculations, they may or may not be realistic. Here, we take a different approach by re-analyzing several published datasets with some of the approaches discussed here, with analysis results compared in the Results section.

## 2. Materials and Methods

Early-on, systematic investigations of interaction effects of two disease-causing variants were carried out by subdividing the data based on genotypes in one gene and then analyzing the resulting two or more groups of data separately for genotypes at other genes. For example, sibpairs affected with diabetes were subdivided into two groups depending on whether or not the siblings shared two *HLA* alleles (on chromosome 6), and only the sharing group exhibited linkage to the *FGF3* gene (on chromosome 11) [14], which provided evidence for gene–gene interaction between *HLA* and *FGF3*. The principle of subdividing data and analyzing each portion separately has been formalized as a sequential procedure in genome-wide association studies [15]. Rather sophisticated data mining approaches like CART and SVM also make use of subdivisions of data [16]. Combined effects of multiple susceptibility variants have been investigated by summing over single-locus test statistics and treating such a sum as a single test statistic. Sums over lod scores in linkage analysis [17] or over test statistics in case-control association analysis [18] have been constructed, but only the latter furnish empirical significance levels associated with results. Here, we discuss a selection of methods we consider particularly relevant for current machine learning approaches in case-control studies.

Multifactor dimensionality reduction (MDR): In human genetics, MDR is the first machine-learning approach for detecting gene-gene interactions and has been highly successful in the 20 years since its inception [19,20,21]. It has been applied to various diseases, e.g., breast cancer [22]. For given *n* variants and their interaction effects on disease, MDR focuses on the *n*-dimensional array of genotypes. With 3 genotypes per variant, the total number of genotype arrays (genotype patterns, also called diplotypes) is given by 3^n^. Each pattern is classified as low-risk or high-risk depending on a threshold ratio of case versus control individuals carrying that pattern so that, effectively, the analysis problem is reduced from *n* dimensions to one dimension [11]. Various statistical techniques like cross-validation are then carried out to optimize prediction accuracy of individuals being classified as cases or controls. Resulting patterns (“models”) are ranked based on overall balanced accuracy [23], that is, a balance between high power and low *p*-value. As recommended [21], we built a permutation framework around MDR (see Supplementary Materials) so that we can assign empirical significance levels (*p*-values) to each of the best patterns (“Top Models”), where the null hypothesis is absence of association between disease phenotype and variants [21].

Advantages of MDR include the availability of a mature and easy to use software, the model-free approach, and that the number of interaction terms does not grow exponentially as new variables are added [11]. On the other hand, high rates of phenocopies and genotyping errors tend to greatly reduce power [20], but that may be true of many other methods as well. Using multiple CPUs for parallel processing, MDR can run large numbers of variants.

Average effects of SNPs: For *n* SNPs and corresponding 3^n^ genotype patterns, an alternative way of dimension reduction is based on the observed joint effect of SNPs as follows [24]: For a given genotype pattern, *k*, define *t*_k_ = (*n*_D,k_/*n*_D_ − *n*_U,k_/*n*_U_)^2^, where *n*_D,k_ and *n*_U,k_ are respective counts of cases and controls with the genotype pattern, and *n*_D_ and *n*_U_ are the respective total numbers of cases and controls in the study. The joint effect of the *n* SNPs is then defined as the sum of *t*_k_ over all patterns, *k* = 1, …, 3*^n^*. This method incorporates main and interaction effects, and permutation testing has shown significant results for breast cancer genes, for which marginal effects were non-significant [24]. No software is available incorporating this method.

AprioriGWAS: This method is based on frequent pattern mining (FPM), also called Frequent Itemset Mining (FIM) [25]. FPM originated with the Apriori algorithm [26], which was designed to accommodate the huge and ever increasing databases obtained at cash registers recording items (goods) purchased by consumers. The collection of items bought by a consumer was called an itemset and each consumer represented a transaction. An important feature of the Apriori algorithm is its ability to generate association rules, that is, conditional probabilities (predictions) that customers tend to buy a specific item like wine given they purchase some other items like bread and milk.

Based on the Apriori algorithm, AprioriGWAS [27] was designed to find disease-associated variants through their interaction effects. Applied to Bipolar Disease, AprioriGWAS found significant interactions among variants without significant main effects. Developed less than ten years ago, this approach represents a new way of investigating multi-variant association analysis. Its main advantage is its strong reliance on FIM methods in genetic disease association studies, and it shares with MDR the ability to find epistatic interactions in the absence of single-variant main effects. On the other hand, its focus on testing for gene–gene interactions necessitated the development of a new “conditional permutation” approach [27]. This publication [27] contains applications to datasets on age-related macular degeneration (AMD) [28] and to bipolar disease in the WTCCC data collection [29], and found respective 166 and 200 disease-associated pairs of interacting variants. The two datasets contain 96,607 and 393,271 SNPs, respectively. Analysis of those numbers of SNPs required huge computing resources and was carried out in a cluster of 1000 CPUs. A software package with instructions is available, although it may not be easy to use.

This new approach seems to have spawned six other new methods, all of which quote AproriGWAS: A step-wise approach based on Cochran–Mantel–Haenszel (CMH) statistics [30], ancGWAS [31], FHSA-SED [32], GeDI [33], Epi-GTBN [34], and EpiMOGA [35], where the latter presents a nice, brief overview of epistasis detection methods. Two of these new methods are discussed below, namely Stepwise CMH and Epi-GTBN.

Stepwise CMH: The Cochran–Mantel–Haenszel (CMH) test is a classical statistical procedure for analyzing multi-way contingency tables [36]. In the Stepwise CMH approach [30], a new variant is added to an existing set of variants based on the CMH test. To reduce the burden of dimensionality, the sum of minor allele counts (MACs) is used to stratify data, thus effectively working along a single dimension. The method performs forward steps, adding the most disease-associated variant, and backward steps, eliminating the least significant variants, and terminates until no more significant variants are added. The computational resources required seem to be lower than those for AprioriGWAS. The Stepwise CMH method was also applied to the Bipolar Disease WTCCC dataset [29] and resulted in a set of 16 disease-associated SNPs, one of which, rs11984645, was also found to be highly significant by AprioriGWAS in the same dataset. Software for the Stepwise CMH method is available as R code [30] (http://bibs.snu.ac.kr/software/stepCMH/ (accessed on 28 July 2021)).

Epi-GTBN: Like many other approaches, Epi-GTBN [34] employs a Bayesian network, that is, a probabilistic model to represent actions and interactions among variants and phenotypes. To optimize network parameters and minimize the chance for local optimization, a special form of a genetic algorithm is applied to iteratively optimize model parameters. In addition to computer-generated datasets, authors also analyzed a well-known dataset on AMD [28], which has been investigated by various other researchers. For analysis by Epi-GTBN, to reduce the computational burden, only the 1039 SNPs with smallest *p*-value (*p* < 0.01) out of the original 103,611 SNPs were retained (the corresponding statement in [34] must be an error). Results obtained by Epi-GTBN and comparisons with other methods are shown below.

GPM: Genotype pattern mining (GPM) represents a modification of AprioriGWAS with a simpler structure and more straightforward theoretical basis. The aim here is not to detect epistasis but to test for frequency differences of genotype patterns between cases and controls. As is well-known, variants in close proximity to recessive or dominant traits tend to be more homozygous [37] or heterozygous [38], respectively, than when they are elsewhere in the genome, which is the basis for genetic association mapping. Here, we extend this notion from variants to variant pairs, specifically, from genotypes to genotype pairs (patterns), where genotypes are labeled 1, 2, and 3 for AA, AB, and BB, respectively.

For given two variants at different genomic locations, consider a specific genotype pattern, for example, X = (2, 3), that is, the genotype is 2 = AB at variant 1, and 3 = BB at variant 2. For each variant, we set up a 2 × 2 table with rows corresponding to cases and controls, and columns for “X present” and “X absent” (Table S1 in Supplementary Materials). We compute a suitable statistic (the likelihood ratio chi-square) to test the null hypothesis of no association between phenotype and genotype pattern. For case-control data genotyped at a number of variants (SNPs), we employ a general-purpose FIM algorithm, fpgrowth [39,40], as our core engine and ask it to find all two-locus genotype patterns with minimum values for support (pattern frequency in the data) and confidence (proportion of cases with the pattern) [26].

For each of the potentially large number of detected digenic genotype patterns in cases and controls, we compute chi-square as a measure for the discrepancy in pattern frequency between cases and controls. To address the multiple testing problem inherent in this approach, we implemented a straight permutation framework to obtain a *p*-value for any genotype pattern, corrected for multiple testing. For reasons outlined below, we focus on patterns of length two, that is, pairs of genotypes from any two variants. In contrast to MDR, which focuses on 3 × 3 contingency tables of genotypes for two SNPs, our unit of observation is the genotype pattern, of which there are 3 × 3 = 9 for a pair of SNPs. In such a table, the 9 genotype patterns represent 8 df (degrees of freedom), which may be partitioned into 2 df for main effects in cases and controls each, and 4 df for interaction/heterogeneity effects (software available).

The starting point for a GPM analysis is a dataset in standard plink format [41,42], and a utility program transforms the data into a format so that GPM recognizes each genotype and phenotype as a separate item. A software package is freely available (https://lab.rockefeller.edu/ott/programs (accessed on 28 July 2021)) for Windows, with a Linux version being in preparation.

## 3. Results

Here, we present results obtained by GPM and some of the other programs discussed above, notably MDR, as this is the most well-known epistasis analysis method in human genetics. As mentioned above, we perform these comparisons on the basis of published datasets rather than theoretical, computer-generated data. Detailed program parameters used for GPM and MDR are provided in Supplementary Materials.

### 3.1. AMD Dataset

This dataset [28] contained 96 cases with age-related macular degeneration (AMD) and 50 controls, each genotyped for 103,611 SNPs. As this number was too high for analysis by GPM, we selected the 2000 SNPs with largest chi-square in the association trend test. MDR requires fully genotyped data, so we further removed 621 variants with missing genotypes for analysis by MDR.

Guo et al. [34] listed 171 two-variant patterns (pairs of SNPs) obtained by the Epi-GTBN Bayesian method, based on the best 1039 SNPs in this dataset (see above). On the other hand, AprioriGWAS [27] reported 166 highly significant variant patterns. Among these two lists of patterns, 17 patterns were shared. This high overlap is a testament to the efficacy of both methods. However, the two lists also contained variants with strong main effects. Of the two significant SNPs in the original AMD study [28], rs380390 and rs10272438, the former occurred in both lists and the latter was picked up only by Epi-GTBN. Therefore, these methods have tendencies to find SNPs with strong main effects while interacting with other SNPs.

Comparing Epi-GTBN with MDR, the latter found 102 variant pairs with *p* < 0.05. The two datasets shared 15 variants, which is comparable to the sharing between Epi-GTBN and AprioriGWAS. Regarding rs380390, the most significant SNP in the original AMD study [28], the variant lists furnished by Epi-GTBN and MDR contained respective 104 (61%) versus 82 (80%) variant pairs containing that SNP. Clearly, these “epistasis-detecting” methods are strongly influenced by variants with main effects.

The three methods compared above focus on variants and their interactions, while GPM works with patterns of genotypes rather than variants. It found 132 genotype patterns with *p* < 0.05, support > 35, and confidence > 90%. Of these patterns, only 70 (53%) contained a genotype in the rs380390 variant, which compares favorably with the two methods above. Next, we compared the SNP pairs, in which the genotype pairs identified by *GPM* occur, with those SNP pairs identified by MD. Surprisingly, there was no overlap between the two sets of SNP pairs. Turning to individual variants, disregarding their occurrence in pairs, the GPM and MDR lists contained 139 and 130 unique SNPs, respectively. Eleven SNPs are shared between the two sets of SNPs. Finally, comparing variant pairs identified by Epi-GTBN with those containing the genotype patterns identified by *GPM*, eight variant pairs were shared between the two sets, still a relatively high number given that Epi-GTBN and GPM are based on very different methodologies.

### 3.2. Opioid Dataset

Some of us previously investigated involvement of eight genes in the opioid system (OPRD1, OPRK1, OPRL1, OPRM1, PDYN, PENK, PNOC, and POMC) and their potential effects on substance abuse [43]. Specifically, we analyzed 82 variants genotyped in 143 opioid-dependent patients in methadone maintenance treatment and 153 healthy controls.

Applying our new GPM method, searching for genotype patterns with a minimum of 5 occurrences (support) in the data, and a minimum proportion (confidence) of 80% of cases among carriers of the pattern, we found only one significant pattern, that is, genotypes (2, 2) (both heterozygotes) in variants rs1918760 on chromosome 1 and rs6136667 on chromosome 20, *p* = 0.022, with observed support and confidence of 14 and 100%, respectively. For an analysis with MDR, missing genotypes had to be eliminated, which can be done by removing offending variants (with parameter –geno 0 in plink) or offending individuals (–mind 0). With the former solution, rs6136667 and 18 other variables were lost, and the latter solution reduced the number of cases and controls by six each. Neither of these reduced datasets furnished significant results with MDR (*p* > 0.78) while the dataset with lower numbers of individuals retained its significance (*p* = 0.030) when analyzed by *GPM*.

To see what might be so special about that single significant genotype pattern, we set up a 2 × 9 contingency table with rows corresponding to the two phenotypes, cases and controls, and the columns representing the 3 × 3 pairwise genotypes at the two variants, rs1918760 and rs6136667. When the resulting 8 df are partitioned into main and interaction effects, the latter are seen to be most significant (Table 1). In the display of 3 × 3 genotype tables for cases and controls each (Table 2), we see that the (2, 2) genotype pattern (heterozygous at each of the two variants) occurs in 14 cases but is completely absent in controls. Evidently, it is this single genotype pattern that separates cases and controls.

### 3.3. Schizophrenia Dataset

For a small pilot study on schizophrenia, 14 cases and 23 controls had been collected in an isolated village in Sardinia, Italy [44]. Individuals were ascertained to be distantly related yet with no common ancestors for at least the past four generations. A total of 255,053 SNPs were available for analysis, after quality control. In single-variant association tests, no variant showed significant disease association after correction for multiple testing.

To allow for processing by GPM and MDR, based on the trend genetic association test, the best 2000 SNPs were selected for digenic analyses. To accommodate MDR’s requirement of no missing genotypes, 134 variants had to be deleted. Both approaches furnished more than 500 significant variant pairs, *p* < 0.05. With *p* < 0.01, GPM (minimum *s* = 12, *c* = 80%) and MDR found 970 and 19 variant pairs, respectively. Five variant pairs are shared between the two resulting lists. The most interesting genotype pattern found by GPM, also identified as the top variant pair by MDR, is (3,3) (two homozygotes for the common alleles) in variants rs2300161 on chromosome 1 and rs7575062 on chromosome 2; the former is in intron 2 of the PTGER3 gene, and the latter resides in a region with regulatory elements (http://rv.psych.ac.cn/variant.do?variant=rs7575062 (accessed on 28 July 2021)). This genotype pattern occurs only in schizophrenics and in none of the controls, that is, it perfectly separates cases from controls. As this was a very small study, further work is required to see whether this finding holds up in larger samples.

## 4. Discussion

As MDR is arguably the most well-known epistasis detection method, we mostly compare our new GPM method with MDR. Both approaches have in common the fact that they can find interacting variants (genotypes in the case of GPM) without resorting to single-variant effects. However, they evidently apply rather different criteria for ranking pairs of variants and genotypes. MDR focuses on 3 × 3 genotype tables, while in the GPM approach, individual pairs of genotypes (of which there are nine per pair of variants) are the unit of observation. Consequently, as seen in the Methods section, results can be rather different. Statistically, MDR focuses on prediction by maximizing its ability to classify individuals into cases and controls, while GPM focuses on statistical significance. We have the well-known conundrum that significant variables are not necessarily good predictors [45], and good predictors might not be significant. Ideally, we want good predictors that are significant so they are not likely to be due to chance, which is why we incorporated MDR into a permutation framework that can judge each variant pattern found.

Our implementation of basic permutation testing [46] will result in proper estimates of empirical significance levels. While genotype or variant patterns are not independent, individuals and their phenotypes are, so randomly permuting individual phenotypes will create proper null samples with no association between phenotypes and genotypes (null hypothesis, H_0_). To some degree, *p*-values depend on program parameters, so searching for small *p*-values by changing parameter values will no longer guarantee a proper significance level, but this situation occurs in many applications of statistical principles. For the limited number of distinct chi-square values obtained from our 2 × 2 tables of phenotype versus presence and absence of a pattern, considerable improvements over classical permutation testing have been made [47] but are not discussed here further.

Some authors advocate searching for higher-order interactions by considering more than two variants at a time, judging approaches based on pairs of variants as being “of limited utility” [35]. While higher-order interactions may well be able to provide insights unavailable on the basis of two-SNP interactions, such analyses may require highly increased sample sizes. Here, we show a telling example demonstrating that *p*-values can be dramatically increased in the search for higher-order interactions, when sample sizes are kept constant. In our opioid dataset discussed above, with minimum support and confidence of 14 and 80%, respectively, searching for genotype patterns of length 2, *GPM* found the genotype pattern (2, 2) in variants rs1918760 (chromosome 6) and rs6136667 (chromosome 20) as the best of all 36 identified patterns, with *p* = 0.019. If instead we search for patterns or lengths 2 or 3, GPM finds a total of 4062 patterns, 36 of which are of length 2. The best of these two-variant patterns is the same as the one found in the search of patterns of length 2, but now the associated significance level is 0.419, and the best pattern of length 3 also occurs with *p* = 0.419. The increase from *p* = 0.019 to 0.419 may be explained by the greatly enlarged statistical sample space, which opens up many more opportunities for random findings. Presumably, a greatly increased sample size would be needed to find significant results for patterns of lengths 2 or 3 if indeed some of these are real. Thus, it seems preferable to confine searches to patterns of length 2 and to suitably combine these to find longer “chains” of interconnected variants.

Currently, GPM is designed to work on qualitative traits. The simplest extension to allowing for quantitative traits would be to dichotomize them or, better yet, to focus on the extreme ends of quantitative trait distributions. Such a design tends to be very powerful [48] and can be accommodated with the current program version. A well-known approach to detecting variants interacting on quantitative traits is the combinatorial partitioning method (CPM) [49]. At this time, we have no immediate plans to extend our method to quantitative traits but may consider this in the future.

## Figures and Tables

**Table 1:** Partitioning of chi-square for the two genotypes in the most significant genotype pattern for the Opioid dataset. The interaction effect is more significant (smallest *p*-value) than either of the two main effects.

Source	Chi-Square	df	*p*
rs1918760 main	2.329	2	0.3121
rs6136667 main	7.388	2	0.0249
interaction	12.592	4	0.0135
Total table	22.309	8	0.0002

**Table 2:** Bivariate distribution of genotypes in the best genotype pattern for the Opioid dataset, separately for cases and controls. As shown in bold, the unique difference between cases and controls is the presence of pattern (2,2) in cases and its complete absence controls. The numbers of the other eight genotype patterns look comparable in cases and controls, so the effect of the (2,2) pattern could be “diluted” when contingency tables rather than genotype patterns are compared between cases and controls.

	rs136667 Genotypes		
rs1918760 Genotypes	*1*	*2*	*3*
**Cases**			
1	0	1	4
2	1	**14**	39
3	1	16	65
**Controls**			
1	0	1	4
2	1	**0**	45
3	1	15	86

## Data Availability

The *fpgrowth* program is available from the website, https://borgelt.net/software.html (accessed on 28 July 2021). The *GPM* program is available at https://lab.rockefeller.edu/ott/ (accessed on 28 July 2021). *MDR* was downloaded from https://sourceforge.net/projects/mdr/ (accessed on 28 July 2021). *AprioriGWAS* is available at https://github.com/Qingrun/AprioriGWAS (accessed on 28 July 2021).

## Supplementary Materials

The following are available online at https://www.mdpi.com/article/10.3390/genes12081160/s1, Table S1: Parametrization of the four types of observations in GPM analysis; technical details of our implementation of *MDR* and *GPM*.
