# Supplementary material for: Genotype Pattern Mining for Pairs of Interacting Variants Underlying Digenic Traits
Source: Genes (Basel). 2021 Jul 28;12(8):1160. doi: 10.3390/genes12081160 (PMC8391494; doi:10.3390/genes12081160)
Supplement: Supplementary file 1 [file genes-12-01160-s001.zip › genes-1292500-supplementary.pdf]

Genotype Pattern Mining for pairs of interacting variants underlying digenic traits

Atsuko Okazaki, Sukanya Horpaopan, Qingrun Zhang, Matthew Randesi, and Jurg Ott

Supplementary Material

The two programs discussed here are Multifactor Dimensionality Reduction (MDR) and Genotype Pattern Mining (GPM). *MDR* has been a mainstay in human case-control epistasis analysis [1, 2]. Below, we describe our implementation of *MDR* for permutation testing [3]. *GPM* has been newly developed and uses a general-purpose FIM algorithm, *fpgrowth* [4], as its pattern search engine. The starting point for each of the two approaches (our implementations) is a dataset in standard *plink* [5, 6] format, that is, as *.*ped* and *.*map* files. Small utility programs can then transform the *plink* files into a format useable by *MDR* and *GPM*. Both programs are currently available for Windows, and a Linux version is in preparation for *GPM*. Each program should be run in a command window (cmd).

MDR, program parameters

The Multifactor Dimensionality Reduction (MDR) program furnishes variant patterns ordered by their Balanced Accuracy Overall (BAO) and lists as "Top Models" those with highest BAO [7]. It also judges patterns by a more complex combination of statistics and will declare a "best model". In practice, the "best" and the "top" models are usually identical. We chose to strictly work with the BAO criterion [8] and built a standard permutation framework around *MDR* so that each variant pattern ("model") can be assigned a significance level. *MDR* has a built-in possibility to carry out permutation testing but a *p*-value will only be issued for the best model, so we do not make use of *MDR*'s permutation feature. Calling *MDR* repeatedly from within our script, we apply the following parameter values:

-min=n_1, -max=n_2, -nolandscape, -top_models_landscape_size=n_3,

where the user furnishes relevant numbers as follows: n_1 = minimum number of variants in a pattern, n_2 = maximum number of variants in a pattern (recommended: $n_1 = n_2 = 2$), and n_3 = number of patterns with highest BAO output by *MDR*. Also specified is the desired number n_P of permutations. Permutations will include the observed data as a null dataset so that the smallest possible empirical significance level will be $1/n_P$. As recommended [7], *MDR* can be downloaded from https://sourceforge.net/projects/mdr/.

GPM

This program package is available from https://lab.rockefeller.edu/ott/programs. It focuses on genotype (rather than variant) patterns and will output genotypes in each pattern found and the variants containing these genotypes. Missing genotypes are allowed but when *fpgrowth* furnishes

patterns containing missing genotypes, these patterns will be intercepted by the *GPM* main program. Like *MDR*, *GPM* can handle patterns of length two or more although patterns containing two genotypes are the default and are recommended (see Discussion in main paper).

Input parameters for *GPM* consist of three numbers, indicating (1) whether pattern frequencies and associated statistics should be obtained for cases or for controls, (2) minimum number of individuals carrying a pattern (support), and (3) minimum proportion of cases among individuals with the current pattern (confidence, in %). Optionally, two additional numbers can specify minimum and maximum lengths of patterns. Recommended minimum confidence is 80% (https://borgelt.net/doc/fpgrowth/fpgrowth.html) and minimum support should be chosen based on the sample size in the data; reasonable values are 5 or 10.

	X pattern	not X	
Y	α	β	N_2
not Y	γ	δ	N_1
	s	$N - s$	N

Supplementary Table S1. Parametrization of the four types of observations in *GPM* analysis.

Hypothesis testing. For a given pattern, X, we want to test its association with a phenotype, Y, here, of being a case (affected by disease). Table S1 shows four types of observations, α = number of cases carrying X, β = number of cases not carrying X, γ = number of controls with X, and δ = number of controls lacking X. The *fpgrowth* program will furnish association rules, R = "$X \rightarrow Y$", with associated observed support, $s = \alpha + \gamma$, and observed confidence, $c = \alpha/(\alpha + \gamma)$, which is an estimate for the conditional probability of being a case, $P(Y|X)$, given an individual carries the X pattern. Thus, we can retrieve the 2 × 2 table above as $\alpha = c \times s$, $\beta = N_2 - \alpha$, $\gamma = s - \alpha$, and $\delta = N_1 - \gamma$, and compute an appropriate test statistic in support of the alternative hypothesis of association between X and Y. We chose the likelihood ratio chi-square as our test statistic.

References

1. Moore JH, Hahn LW. A cellular automata approach to detecting interactions among single-nucleotide polymorphisms in complex multifactorial diseases, Pac Symp Biocomput 2002:53-64.

2. Moore JH, Ritchie MD. STUDENTJAMA. The challenges of whole-genome approaches to common diseases, Jama. 2004;291:1642-1643.

3. Manly BFJ, Navarro Alberto JA. Randomization, bootstrap and monte carlo methods in biology. Chapman & hall/crc texts in statistical science. Boca Raton: Taylor & Francis, 2021, 1 online resource.

4. Borgelt C. An implementation of the FP-growth algorithm. Proceedings of the 1st international workshop on open source data mining: frequent pattern mining implementations. Chicago, Illinois: Association for Computing Machinery, 2005, 1–5.

5. Purcell S, Neale B, Todd-Brown K et al. PLINK: a tool set for whole-genome association and population-based linkage analyses, Am J Hum Genet 2007;81:559-575.

6. Chang CC, Chow CC, Tellier LC et al. Second-generation PLINK: rising to the challenge of larger and richer datasets, Gigascience 2015;4:7.

7. Moore JH, Andrews PC. Epistasis Analysis Using Multifactor Dimensionality Reduction. In: Moore J. H., Williams S. M. eds). Epistasis: Methods and Protocols. New York, NY: Springer New York, 2015, 301-314.

8. Winham SJ, Motsinger-Reif AA. An R package implementation of multifactor dimensionality reduction, BioData Min 2011;4:24.
